# Immunotherapy for Leiomyosarcoma: Current Status and Perspectives

**DOI:** 10.1155/jimr/6614578

**Published:** 2026-05-09

**Authors:** Haoyue Qi, Hanxi Zhang, Qin Wang, Xiaolu Wang, Limei Min, Yingling Zhou, Baorui Liu, Rutian Li

**Affiliations:** ^1^ Department of Oncology, Taikang Xianlin Drum Tower Hospital, Affiliated Hospital of Medical School, Nanjing University, Nanjing, China, nju.edu.cn; ^2^ Department of Oncology, Nanjing Drum Tower Hospital, Affiliated Hospital of Medical School, Nanjing University, Nanjing, China, nju.edu.cn; ^3^ Department of Oncology, Nanjing Drum Tower Hospital, Clinical College of Traditional Chinese and Western Medicine, Nanjing University of Chinese Medicine, Nanjing, China, njucm.edu.cn

**Keywords:** immunotherapy, leiomyosarcoma, soft-tissue sarcomas (STS), tumor microenvironment

## Abstract

Leiomyosarcoma (LMS), one of the soft‐tissue sarcomas (STSs), has a relatively higher risk of distant metastasis. This has resulted in current first‐line treatments still failing to deliver satisfactory outcomes, and the current treatment of LMS remains controversial and challenging. Therefore, it is worth thinking about how to guide and decide on the direction of treatment for LMS. In this review, we analyzed the immune microenvironment, gene expression, and molecular landscape of LMS, hinting at future research directions and prospects for LMS. We also analyzed the classical immunotherapy led by immune checkpoint inhibitors (ICIs), individualized immunotherapy such as cell therapy, and gene editing technologies, which bring better effect mechanisms and survival benefits, as well as opportunities and challenges for large‐scale clinical applications.

## 1. Introduction

Leiomyosarcoma (LMS) is one of the common subtypes of malignant heterogeneous mesenchymal tumors, accounting for approximately 10%–20% of newly diagnosed soft‐tissue sarcomas (STSs) in the U.S. population [1], with an incidence rate (the overall crude incidence rates) of approximately 0.7/100,000/year reported in European cohorts [2]. LMS originates from smooth muscle or its precursor cells and can occur anywhere in the body, often in the retroperitoneum, limbs, and uterus. Based on pathological features, it can be classified into uterine LMS (uLMS) and other types (retroperitoneal, gastrointestinal, limb, and subcutaneous LMS), among which uLMS is the most common type of uterine sarcoma [3].

LMS is characterized by its inherently aggressive biology, with approximately 90% of patients presenting with high‐grade (Grades 2–3) disease, which translates to a significantly higher risk of distant metastasis for the individual patient compared to other histological types of sarcomas [4]. Standard R0 resection remains the cornerstone for patients with localized LMS, regardless of the site of origin, with adjuvant or neoadjuvant radiotherapy used in some cases.

For advanced disease, chemotherapy is the mainstay to delay progression and improve survival. First‐line treatment has traditionally relied on regimens such as doxorubicin, gemcitabine, and ifosfamide [5, 6]. In recent years, a pivotal Phase III trial (NCT02997358) established that combination therapy with doxorubicin and trabectedin, followed by trabectedin maintenance, significantly improves both median progression‐free survival (PFS) and overall survival (OS) compared to single‐agent doxorubicin, representing a major advancement in the first‐line setting [7]. Subsequent therapy options include dacarbazine, temozolomide, anlotinib, or pazopanib [8–10].

However, treatment of LMS still has shortcomings, especially the limited effectiveness of options for advanced/metastatic LMS, where short‐term benefits and overall prognostic improvement are unsatisfactory [11]. Despite the improvement with doxorubicin–trabectedin combination, most patients eventually progress and require further therapy, with limited effective subsequent options [12–15]. The overall therapeutic outcome for advanced LMS remains far from satisfactory. Therefore, against the current treatment landscape, actively exploring novel therapeutic mechanisms such as immunotherapy is crucial for breaking through the existing efficacy limitations in LMS.

**Figure 1:**
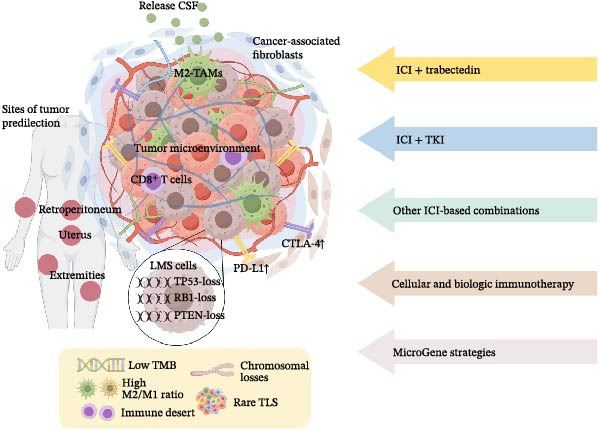
Schematic overview of the immune microenvironment in LMS and current emerging immunotherapeutic strategies.

Since the 19th century, when William Coley treated sarcoma with intratumoral injections of modified bacteria, immunotherapy has grown exponentially over the course of more than a century. Immune checkpoint inhibitors (ICIs) represent a major advancement in modern immunotherapy for solid tumors, offering new perspectives both as single agents and in combination with other drugs and therapies [16–18], while the field has spawned more new directions, such as tumor vaccines [19], adoptive cell therapies (ACTs) [20, 21], and so on. However, the complex karyotype, low tumor mutation burden (TMB), and low apoptotic fraction of LMS still result in variable treatment responses, suggesting a poor CD8^+^ T cell infiltration. Figure 1 shows the immune microenvironment in LMS and current emerging immunotherapeutic strategies. This review aims to critically evaluate the immune microenvironment of LMS, summarize clinical experience with immunotherapy, and highlight future research priorities.

## 2. Immune Microenvironment and Gene Expression in LMS

### 2.1. T Cell

A microenvironmental cell population analysis of data from 608 STS subtypes classified STS according to the immune microenvironment, with LMS mostly classified in the “immune desert” (SIC A) and hypoimmune groups (SIC B) [22], suggesting poor CD8^+^ T cell infiltration. This scarcity of cytotoxic T cells deprives ICIs of their primary effector population, explaining why programmed cell death protein 1/programmed death ligand 1 (PD‐1/PD‐L1) blockade often fails in LMS. Similarly, Klaver et al. [23] suggested that CD8^+^ T cells are low in LMS compared to other STS, with approximately one‐third of LMS displaying a “CD8^+^ T cell‐poor” phenotype. LMS showed a higher proportion of T_EM_ cell and CD45RA effector memory T (TEMRA) cells and a significantly lower proportion of regulatory T cells (Tregs). The immune microenvironment of LMS has a high abundance of fibroblasts and a low abundance of T cells. Thus, merely increasing T cell abundance without dismantling the fibroblast‐built physical and cytokine barriers may be insufficient to restore ICI sensitivity [24].

In terms of antigen presentation, LMS has higher expression levels of *HLA-A*, *HLA-B*, *HLA-C*, *HLA-G*, and *HLA-DPB1* genes compared to other STS subtypes and also among related to T cell genes. The expression of T cell infiltration marker CD3D, positive T cell markers CD8A and CD127, and mediated to T cell inflammation‐related molecule IL‐2RA are all elevated in LMS [25]. This paradox—high *HLA* and T cell signature gene expression yet sparse intratumoral T cells—implies that antigen presentation machinery is intact but immune exclusion dominates, suggesting that therapeutic strategies should focus on T cell trafficking and retention rather than further boosting antigen presentation. Despite these indications of immunogenicity, functional T cell infiltration remains limited. This limitation is associated with immunosuppressive molecules, particularly the highly expressed key regulator IDO. PD‐L1 positivity in LMS varies considerably across studies and anatomical sites: 5.8% of 69 LMS cases showed tumoral PD‐L1 expression in a 5,536‐case cohort, whereas smaller dedicated series report substantially higher rates [23, 25–27]. Collectively, these data indicate that PD‐L1 positivity alone is an inadequate biomarker for ICI response in LMS; combination metrics integrating T cell density, fibroblast burden, and IDO activity are urgently needed.

### 2.2. B Cell and Tertiary Lymphoid Structures

TLSs are also a very important component of the tumor microenvironment (TME). TLS are immune cell aggregates that form in nonlymphoid tissues under nonphysiological conditions later in life. B cells selectively activate and expand in the germinal centers of mature TLS and differentiate into plasma cells to further exert their role after undergoing antibody class switch and somatic hypermutation. The presence of TLS and B cells correlates with better survival and response to immunotherapy [28–30]. TLS is uncommon in LMS, with TLS‐positive LMS accounting for approximately 12.2% of LMS [31], and the few distinct subgroups of LMS that possess TLS are of the immunologically active type, which also appears to have a durable response to ICIs [32].

In a Phase II trial combining pembrolizumab with low‐dose cyclophosphamide for advanced STS, TLS‐positive patients exhibited markedly improved 6‐month nonprogression rates (40% vs. 4.9%) and objective response rates (ORRs; 30% vs. 2.4%) compared to the overall cohort [31]. Thus, if mature TLS or high‐abundance plasma cells are found in the tumor immune microenvironment of LMS patients, this group may derive significant benefit from ICIs as single agents and is likely to benefit from combination immunotherapy. In addition, the induction of de novo TLS in some immunocompromised phenotypes is also a promising direction for future immunotherapy [33].

### 2.3. Tumor‐associated Macrophages (TAMs)

TAMs influence tumor cell development, stromal lysis, immune promotion and suppression, angiogenesis, and other processes [34]. Undifferentiated macrophages (M0 TAMs) are polarized into M1 or M2 TAMs under the stimulation of various cytokines [35]; M2 TAMs, which express CD206 and CD163, promote tumor immune escape and metastasis; while M1 TAMs, which express CD86 and CD80, are considered tumor‐killing macrophages and exhibit antitumor and immune‐promoting effects [36]. CD163+ macrophages tend to move toward the M2 phenotype in the presence of macrophage colony‐stimulating factor (M‐CSF) produced by LMS [37]. While TME of LMS suggests substantial macrophage infiltration, the antiphagocytic signal from tumor cell CD47 interacting with macrophage receptor signal‐regulating protein α (SIRPα) further promotes LMS growth [38]. Interestingly, in LMS with wide variation in TAM infiltration levels, increased density of CD68^+^ or CD163^+^ macrophages tended to correlate with poor outcomes in nongynecologic LMS, without significant differences in uLMS [39], and Kostine et al. [37] also confirmed the possibility that M2 TAMs are associated with a poor prognosis in LMS. CD163^+^ and CD204^+^ TAMs growing concurrently in the tumor margin zone tend to correlate with short disease‐free survival (DFS) in patients [40]. Although formal demonstration in LMS is still lacking, the same immunosuppressive axis—high CSF1‐driven TAM infiltration, IL‐10/TGF‐β secretion, and PD‐L1 expression—has been shown in other solid tumors to blunt anti‐PD‐1 efficacy [41]. Given that LMS releases abundant CSF1 and harbors CD163^+^/CD204^+^ TAMs, it is plausible that this mechanism also lowers ICI response.

### 2.4. Tumor Gene Expression and Molecular Landscape

TMB is broadly defined as the number of somatic mutations in each coding region of the tumor genome. TMB is thought to be a key driver of the generation of immunogenic neopeptides displayed on the major histocompatibility complex (MHC) on tumor cells, allowing the immune system to differentiate between normal and tumor cells, which affects T cell activity and consequently the patient’s response to ICI [42, 43].

TMB is particularly critical in LMS with high heterogeneity, and an analysis of 1 million human cancer genomes suggests that the median TMB for LMS is around 2.5 mut/Mb, with TMB exceeding 20 mut/Mb in only 0.7% (non‐uLMS) and 0.9% (uLMS) [23, 44].

LMS demonstrates profound genomic complexity, characterized by recurrent copy‐number alterations, chromosomal instability, and mutational heterogeneity—features that collectively undermine immunotherapy responsiveness. Among these, frequent changes in the damage pathway of tumor suppressor genes, such as *TP53*, *RB1*, and *PTEN*, can be considered hallmarks of LMS. Biallelic inactivation of *TP53* and *RB1* is very common. High expression of genes related to myogenic differentiation, such as *Mylk*, *MYH11*, *ACTG2*, *miR-143*, and *miR-145*, is accompanied by various uncommon mutant genes and widespread somatic copy‐number alterations, with recurrent loss of chromosomes 10, 11q, 13, 16q, and 17p13 regions, and recurrent gain of chromosome 17p12. Other variable patterns of localized chromosome shattering further increase the genomic complexity of LMS. And overcoming replication mortality occurs primarily by alternative lengthening of telomeres [45–47]. The PI3K/AKT pathway is frequently overactivated in either non‐uLMS or uLMS [48], a phenomenon often caused by loss of *PTEN*, a negative regulator of the PI3K pathway. This not only exacerbates tumor cell proliferation but also inhibits apoptosis. *PTEN* loss also appears to correlate with PD‐1 expression [49], an observation that has been validated in other tumors [50–52]. Notably, LMS exhibits a typical parent–child evolutionary relationship, with common clonal drivers and additional clonal mutations between matched primary and metastatic tumors, with no clearly identifiable metastasis‐related recurrent changes [53].

## 3. ICI Therapy for LMS

While immunotherapy has emerged as a promising therapeutic paradigm for LMS, ICI monotherapy has demonstrated limited clinical efficacy. In a patient with multiple metastases after LMS surgery, where multiple lines of chemotherapy and radiotherapy failed to halt progression, significant lesion reduction was observed with nivolumab alone [54]. However, subsequent Phase II and III results of pembrolizumab monotherapy for LMS were unsatisfactory, with the majority of patients being nonresponsive and a lack of prognostic markers [55, 56]. This suggests that a single immunologic approach is insufficient for LMS. Therefore, more studies have explored combinations of multiple therapeutic modalities, including combinations of immunologic agents or with chemotherapy and antiangiogenic agents, which appear to promise better outcomes [57–59]. Table 1 shows the current relevant clinical studies in LMS.

**Table 1:** The current relevant clinical studies in leiomyosarcoma.

Clinical trials status	ClinicalTrials.gov identifier	Phase	Study start–primary completion	Line of therapy	Enrollment	Drug or drug combination	Patient population	Primary endpoint
Recruiting	NCT06849986	II	2025	—	45	Tislelizumab + liposomal doxorubicin + ifosfamide	Metastatic/locally advanced unresectable sarcoma	ORR
Recruiting	NCT03138161	I and II	2017	≥1	250	Trabectedin + ipilimumab + nivolumab	Metastatic/locally advanced unresectable STS	MTD
Recruiting	NCT04577014	I and II	2020	0	98	Retifanlimab + gemcitabine + docetaxel	Metastatic/locally advanced and unresectable high‐grade STS	24‐week progression‐free survival rate (PFSR)
Recruiting	NCT06528769	II	2025	—	16	All‐trans retinoic acid + cemiplimab	Metastatic/locally advanced unresectable LMS	ORR
Recruiting	NCT06074692	II	2023	≥1	86	Camrelizumab + fluzoparib + stereotactic body radiotherapy (SBRT)	High‐grade sarcoma of bone or soft tissue	6‐month PFSR
Recruiting	NCT03548428	II	2020	< 3	103	SBRT + atezolizumab	Metastatic STS	6‐month PFS
Recruiting	NCT04673942	II	2021	—	140	AdAPT‐001+ICI	Advanced malignant solid tumor	DLT, MTD, safety, and antitumor activity
Completed	NCT02301039 [55]	II	2015–2020	≥1	144	Pembrolizumab	Unresectable, recurrent, and/or metastatic high‐grade soft‐tissue or bone sarcomas	ORR
Completed	NCT02815995 [60]	II	2016–2024	≥1	57	Durvalumab + tremelimumab	Metastatic/progressive sarcoma	PFS
Completed	NCT03899805 [61]	II	2019–2024	≥1	57	Pembrolizumab + eribulin	Liposarcoma, LMS, or undifferentiated/unclassified pleomorphic sarcoma	12‐week PFS
Completed	NCT04242238	I	2020–2025	≥1	32	Avelumab + DCC‐3014	Advanced/metastatic sarcomas	MTD and ORR
Completed	NCT05080790	II	2021–2025	≥1	7	Dinutuximab beta + zoledronic acid + interleukin‐2	Locally advanced/unresectable or metastatic LMS	Feasibility rate
Completed	NCT03123276	I and II	2017–2022	—	24	Pembrolizumab+ gemcitabine	Undifferentiated pleomorphic sarcoma /LMS	DLT and MTD
Completed	NCT00062868 [62, 63]	I	2014–2020	—	74	LMP1/2 CTLs	Epstein–Barr Virus‐associated diseases including EBV^+^ LMS	DLT
Completed	NCT01447056	I	2015–2020	—	37	LMP‐specific T cells	Epstein–Barr virus‐associated diseases including EBV^+^ LMS	DLT
Active, not recruiting	NCT03851614	II	2019	—	90	Durvalumab + olaparib/cediranib	Locally advanced, or metastatic colorectal cancer, pancreatic adenocarcinoma, or LMS	Changes in genomic and immune biomarkers
Active, not recruiting	NCT04095208	II	2020	≥1, <3	67	Nivolumab + relatlimab	Advanced nonresectable/metastatic STS	6‐month PFS
Active, not recruiting	NCT04551430	II	2021	≥1, <3	105	Cabozantinib + nivolumab + ipilimumab	Unresectable or metastatic STS	ORR
Active, not recruiting	NCT05836571	II	2023	—	66	Ipilimumab + nivolumab ± cabozantinib	Metastatic STS	PFS
Active, not recruiting	NCT03282344 [64]	II	2017	≥1	88	Nivolumab + NKTR‐214	Metastatic and/or locally advanced sarcoma	Number of patients with a response
Active, not recruiting	NCT04784247	II	2021	≥1, <3	52	Lenvatinib + pembrolizumab	Metastatic and/or unresectable STS	ORR
Active, not recruiting	NCT04420975	I	2020	—	14	BO 112 + nivolumab + radiation + surgical resection	STS of the extremity, trunk, or retroperitoneum	Incidence of adverse events (AEs)

Currently, ICI–based therapy is undoubtedly one of the more researched and mature categories. Nevertheless, the overwhelming majority of available evidence derives from small, single‐arm trials [55, 59, 65], underscoring the urgent need for adequately powered, randomized controlled studies.

### 3.1. Combined Application of Multiple ICIs

ICIs such as PD‐L1 and cytotoxic T lymphocyte antigen‐4 (CTLA‐4) have changed the paradigm of treatment for many cancers, demonstrating improved survival benefits [66–68].

One of the more frequent applications is ipilimumab. The Alliance A091401 trial demonstrated that ipilimumab plus nivolumab in STS—including the LMS subset—was better than nivolumab monotherapy, which may be related to ipilimumab increasing T cell activation, synergizing with nivolumab to enhance T cell reactivity. This trial confirmed that dual checkpoint inhibition can yield durable benefit in at least a subset of LMS cases, with partial responses (PRs) observed in both uLMS and non‐uLMS patients, and one complete response (CR) in uLMS [57].

Similarly, ipilimumab in combination with CX‐072 (a PD‐L1 inhibitor; NCT03013491) achieved promising results in patients with advanced solid tumors, with one LMS patient observed to be in PR for over 12 months [69].

Likewise constructed as a CTLA‐4 plus PD‐L1 blockade, durvalumab plus tremelimumab was tested in a cohort labeled “vascular tumors” (five LMS and five angiosarcomas) [60]. However, this study showed limited activity, with an overall 12‐week PFS of 49% and a median PFS of 1.8 months; crucially, the specific outcome for LMS remains unreported, and the small sample size precludes definitive conclusions for this histology [70]. Because durvalumab plus tremelimumab was administered at a reduced tremelimumab dose and predominantly in the heavily pretreated setting, further investigation of this combination is warranted in treatment‐naïve or earlier‐line LMS populations, with prospective selection based on PD‐L1, TMB, or tumor‐infiltrating lymphocyte (TIL) density to identify patients who may derive long‐term disease control.

The potential of other immune checkpoints is also being actively investigated. Lymphocyte activation gene‐3 (*LAG-3*) is expressed on CD4^+^ and CD8^+^ T cells and competitively binds to MHC II with CD4, inhibiting T cell proliferation and inducing Treg cell activation [70]. *LAG-3* is emerging as one of the promising targets for cancer immunotherapy in the PD‐1/CTLA‐4 era. A Phase II clinical trial that combined nivolumab (PD‐1 inhibitor) and relatlimab (*LAG-3* inhibitor) for advanced/metastatic STS is currently active (NCT04095208).

### 3.2. Combination of ICIs and Antiangiogenic Agents

Tumor angiogenesis‐promoting immune barriers have been demonstrated, and an increasing number of immune cells exhibit dual ability to promote both immunosuppression and angiogenesis [71]; thus, the combination of antiangiogenic drugs plus ICIs acts synergistically.

Tyrosine kinase inhibitors (TKIs) remodel the vasculature and the immune microenvironment, normalizing the tumor‐abnormalized vascular system [72]. This vascular normalization increases the infiltration of immune effector cells into tumors [72]. Furthermore, the combination of TKIs and ICIs significantly increases the antitumor/protumor immune cell ratio and inhibits immune checkpoint expression more than PD‐1 inhibitors alone, resulting in a more comprehensive immunomodulatory effect [73]. Clinical studies have demonstrated the antitumor activity of ICI–TKI combinations in advanced sarcoma, though LMS–specific data remain limited. A single‐center retrospective analysis of ICIs–TKIs for advanced sarcoma demonstrated the antitumor activity of the combination, while validated biomarkers are urgently needed to predict clinical outcomes; notably, among 20 LMS patients, although the response rate in the combination group was modest, progression was more rapid in the ICI monotherapy group [74]. Although LMS patients were not enrolled, sunitinib in combination with nivolumab showed promising activity in selected patients with advanced STS, with almost half of the patients progression‐free at 6 months [75], suggesting potential applicability in LMS. Similarly efficacious with limited LMS patients included were the combinations of anlotinib or axitinib with PD‐1 inhibitors [59, 76]. Studies such as cabozantinib in combination with PD‐1 inhibitors and CTLA‐4 inhibitors for metastatic STS (NCT04551430) are still ongoing.

Retroperitoneal LMS is often not amenable to radical surgical resection due to proximity to vital organs, and chemotherapy offers very limited benefit [77]. In a retrospective cohort of 57 retroperitoneal sarcomas, eight patients with LMS treated with anlotinib plus camrelizumab achieved a 12.5% ORR and 50% disease‐control rate (DCR) without Grades 3–4 immune‐related toxicity, indicating an expandable targeted immunotherapy pathway for this disease [78].

Notably, patients with somatic mutations such as *PTEN* loss may have better efficacy with ICI–TKI combinations, which may be related to *PTEN* deletion leading to *PI3K* activity, promoting VEGF expression by endothelial cells [79]. Antiangiogenic TKIs may synergize with ICIs by reversing VEGF‐mediated immune suppression and enhancing T cell trafficking into tumors. A case of advanced LMS with biallelic *PTEN* loss achieved a CR with preoperative adjuvant anlotinib and pembrolizumab [80]. However, whether this response requires the combination or could be achieved with TKI monotherapy remains to be determined.

### 3.3. Combination of ICIs and Chemotherapy

Chemotherapy‐induced immunogenic cell death synergizes with immunotherapy. In a clinical trial of doxorubicin plus pembrolizumab, the combination did not meet the ORR endpoint, yet PFS and OS were superior to historical controls, and efficacy was independent of tumor cell PD‐L1 expression [81]. This “PD‐L1–OS disconnect” is common across solid tumors: Even when PD‐L1 is low, sufficient T cell priming can restore cytotoxicity once PD‐1 inhibition is applied. In the same regimen, four PRs and six prolonged stable diseases (SDs) were observed among 10 uLMS patients, yielding a 100% DCR and further confirming clinical benefit despite PD‐L1 negativity [82]. Collectively, the doxorubicin–pembrolizumab regimen shows biological activity in LMS, but larger molecularly annotated cohorts are required to confirm durable efficacy and to develop predictive algorithms beyond PD‐L1. Doxorubicin in combination with toripalimab [83] or sintilimab [84] has involved small LMS subgroups, with some patients achieving SD. It is worth noting that this relatively favorable outcome is limited, and not every effective single‐agent chemotherapy regimen will achieve the expected survival benefit when used in combination [85, 86].

A noteworthy therapeutic strategy is the SAINT study evaluating ipilimumab plus nivolumab combined with trabectedin. Trabectedin, a marine‐derived tetrahydroisoquinoline alkaloid, enhances tumor immunogenicity by affecting the TME and decreasing TAMs and may act synergistically in combination with PD‐1 inhibitors (NCT03590210), while leading to selective depletion of monocytes/macrophages accompanied by a decrease in angiogenesis [87]. The superior efficacy of ICI plus trabectedin compared with other immunological pairings may stem from its dual mechanism: Trabectedin not only exerts direct cytotoxicity but also remodels the immune microenvironment by depleting suppressive macrophages and downregulating angiogenic factors, thereby lowering barriers to T cell infiltration that frequently limit ICI activity in LMS.

In LMS patients, the subtype with the highest enrollment in the SAINT study, a DCR of 89.5%, a median OS of 36.1 months, and an ORR of 31.6% were achieved [88, 89]. These findings warrant cautious interpretation in light of the single‐arm design. Trabectedin is conventionally positioned as second‐line therapy following anthracycline failure, supported by randomized evidence suggesting limited efficacy of first‐line monotherapy relative to doxorubicin‐based regimens [90]. While the SAINT regimen’s median PFS (6.7 months) remains below that of the doxorubicin–trabectedin combination in LMS‐04 (12.2 months) [7], the triple therapy offers a favorable safety profile, circumventing the substantial toxicities (97% Grades 3–4 adverse events) associated with intensive chemotherapy backbones. Thus, this immunotherapy–chemotherapy combination represents a potentially feasible and tolerable first‐line option for selected patients, though prospective randomized validation remains essential. Complementing the SAINT first‐line findings, trabectedin plus avelumab has shown comparable activity in later‐line settings, with a 6‐month PFS rate of 52% reported in advanced LMS and liposarcoma [91].

Other examples of chemotherapeutic agents combined with ICIs, such as nab‐paclitaxel plus sintilimab, need validation in randomized controlled clinical trials [92]. The 12‐week PFS in an LMS cohort treated with eribulin plus pembrolizumab did not reach the expected threshold of >60% [93]. The less frequently used TAS‐116 (pimitespib, an oral HSP90 inhibitor) combined with nivolumab also yielded an objective tumor response in one LMS patient, but further refinement of treatment regimens based on larger patient enrollment is required [94].

### 3.4. Combination of ICIs and Radiotherapy

Radiotherapy is widely used as a neoadjuvant or postoperative adjunct to radical surgery to lower the risk of recurrence. Although the SU2C‐SARC032 study did not include LMS patients, it demonstrated in other STS subtypes that adding pembrolizumab to preoperative radiotherapy and surgery improves short DFS [95]. Beyond local control, irradiation reshapes the immune microenvironment: It upregulates MHC‐I and cancer–testis antigens, increases the secretion of perforin and granzyme B, and promotes a homogeneous antigen landscape that facilitates tumor surveillance [96]. Because LMS exhibits poorer baseline CD8^+^ T cell infiltration than most other STSs, this immune‐priming effect makes radiotherapy an attractive strategy to enhance antigenicity and sensitize tumors to ICIs [97, 98]. In some patients, these changes culminate in an abscopal response, providing systemic protection outside the radiation field [99, 100].

When a patient with metastatic mediastinal LMS was treated with nivolumab and local radiotherapy for 2 months, significant tumor regression was observed in both irradiated and distant metastatic lesions, with an increase in circulating lymphocytes indicative of an activated immune response [97]. The response of each sarcoma subtype to radiotherapy varies, as do its radiosensitivity and changes in the immune microenvironment components. Currently, fewer LMS patients are treated with ICI–radiotherapy combinations. Ongoing clinical trials include NCT03548428, NCT04420975, and so on. Details such as the timing and intervals of radiotherapy and immunologic drug administration need further exploration. Radiotherapy has a bidirectional nature of promoting and suppressing the tumor immune response, which correlates with the total radiation dose and fractionation of radiotherapy [101], necessitating exploration of different dosing schedules.

## 4. Alternative Immunotherapy for LMS

### 4.1. ACT

ACT represented by chimeric antigen receptor T cells (CAR‐T), has almost changed the course of therapeutic development of hematologic malignancies [102, 103], its application in solid tumors has made many rapid advances [104–106], and ACT has attracted much attention due to its features such as high specificity and low drug resistance. Therapeutic attempts of ACT in LMS were also relatively early. As early as 1993, a patient with gastric LMS metastatic to the liver was treated with cyclophosphamide and autolymphocytes, which were mainly composed of tumor‐specific CD45RO+ T cells, and the tumor obviously subsided [107]. The main types of ACT are TIL therapy, CAR‐T cell therapy, and engineered T cell receptor‐T cell (TCR‐T) therapy.

ACT relies on the transfer of autologous or allogeneic tumor‐specific T cells to mediate killing of autologous tumors. More than half of LMS patients can successfully amplify functional TILs [108], and a small percentage have high and uniform levels of TILs [109], holding promise for TIL therapy in LMS after further benchmarks related to TIL efficacy and clinical significance are established.

Cytokine‐induced killer (CIK) cells are a heterogeneous lymphocyte population consisting mainly of CD3+CD56+ natural killer (NK) cells and CD3+CD8+ cytotoxic T lymphocytes (CTLs). CSPG4‐specific CAR–CIK cells prepared with a retroviral vector encoding a second‐generation CSPG4‐specific CAR (CSPG4–CAR) demonstrated superior antitumor activity through tumor recruitment, infiltration, and mechanistic penetration, significantly inhibiting tumor growth in various STS transplantation models. Tumors in two of four LMS mice completely regressed without apparent toxicity [110]. Similarly, TCR‐T therapy holds promising potential for LMS. Investigators constructed *HLA*‐
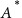
24:02‐restricted, *MAGE-A4*‐siTCR gene‐modified T cells by transducing a codon‐optimized *MAGE-A4* sequence while simultaneously knocking down endogenous TCR expression with siRNA. Significant tumor growth inhibition was observed in NOD‐SCID mice. In a patient with uLMS who achieved CR after four cycles of Endostar, doxorubicin, and gemcitabine, a single infusion of *MAGE-A4*–siTCR‐T cells sustained CR for 31 months, with tetramer‐positive CD8^+^ T cells detectable for >600 days and TCR transgene copies persisting up to day 233. Although the contribution of delayed chemotherapeutic effects cannot be excluded, the long‐term survival and functional persistence of engineered T cells provide encouraging evidence for TCR‐T application in LMS [111].

ACT has strong potential in combating STS such as LMS, and preclinical results show encouraging efficacy. It is believed that it will become increasingly prevalent in oncology and also requires further optimization measures such as gene transduction/editing, improving immune cell plasticity and durability, inhibiting off‐target effects, and discovering more generic tumor‐specific targets.

### 4.2. Cytokine Therapy

Cytokines mediate interactions between immune and nonimmune cells in the TME and are involved in multiple steps of the tumor immune cycle, influencing tumor progression or suppression. Preclinical studies have demonstrated the antitumor properties of several cytokines, with most clinical studies focusing on melanoma, renal cell carcinoma, glioblastoma, and lymphoma [112–114]. Cytokines are generally far less effective as monotherapy than in combination with other antitumor treatments.

IL‐2 was the first approved immunotherapy for cancer [115], but its stimulatory effects on Tregs and dose‐related toxicities have so far prevented the emergence of widely approved IL‐2‐related drugs. Among these, the immunocytokine 14.18–IL‐2 targeting GD2 has demonstrated potential benefit in melanoma and neuroblastoma [116–118]. In uLMS, immunohistochemical analysis showed that nearly two‐thirds of specimens exhibited diffuse strong GD2 positivity, with none characterized as GD2‐negative [119]. Thus, the anti‐GD2 immunocytokine 14.18–IL‐2 may provide a research direction for uLMS. NKTR‐214, a polyethylene glycolated IL‐2 designed to trigger immune cells to attack cancer, is the only compound to have completed a Phase III study. Trials combining it with pembrolizumab for various tumors are ongoing (NCT02799095, NCT04830124, NCT05092360, etc.). A preliminary study in locally advanced/metastatic STS observed remission in one of 10 LMS patients [64].

Other cytokines also have clinical translational research value. Oncostatin M (OSM), a pleiotropic IL‐6 family cytokine, was initially characterized as an antitumor mediator. However, its activation of multiple signaling pathways can also fuel progression in specific tumors [120]. In STS subtypes such as LMS, OSM preferentially enriches CD8^+^ TILs within the tumor compartment without eliciting comparable changes in peripheral blood [121]. Consequently, intratumoral delivery may be the preferred route for OSM, and further studies are required to clarify its in vivo mechanisms and long‐term effects.

### 4.3. Therapeutic Oncology Vaccine

Since William Coley’s use of inactivated *Streptococcus* and *Serratia* for tumor injections [122], the concept of using tumors as antigen reservoirs to enhance immunogenicity through pathways such as tumor antigen release, antigen‐presenting cell maturation, and specific induction of antitumor immune responses has been explored. Some vaccines can induce systemic tumor remission [123], and tumor vaccines represent a new breakthrough in oncology. Many different types of tumor‐associated antigens (TAAs) or tumor‐specific antigens (TSAs) are being studied or developed as the next therapeutic targets for tumor vaccines.

Gangliosides have been extensively researched as “shared” TAAs for some tumors, with related drugs such as the GD2 monoclonal antibody dinutuximab β already approved. The largest clinical trial of a tumor vaccine in sarcoma patients is a Phase II trial of the immune adjuvant OPT‐821 with or without a trivalent ganglioside vaccine against GM2, GD2, and GD3 [124]. Approximately 33% of participants were LMS patients. There was no significant difference in recurrence‐free survival (RFS) between the two groups, with a time to progression of about 6 months and a 1‐year RFS rate of about 35% in each group. This trial may not have prolonged survival because ganglioside expression may be lost in advanced sarcomas, and the trial did not stratify by ganglioside expression. New research strategies focusing on target identification in conjunction with antiganglioside therapy are needed. Additionally, a clinical trial of a dendritic‐cell (DC) vaccine plus gemcitabine in adults and children with sarcoma is ongoing (NCT01803152).

### 4.4. Oncolytic Viruses

Oncolytic viruses, as replication‐competent tumor‐killing viruses, enhance antitumor immunity through the dual mechanism of selectively killing tumor cells and inducing systemic antitumor immunity, and tend to play a more potent role when combined with therapies like ICIs [125]. Dozens of viruses have been used, and genetically engineered viruses that proliferate only in tumor cells offer advantages over wild‐type strains, with herpes simplex viruses being a main representative.

Talimogene laherparepvec (T‐VEC) is an ICP34.5/ICP47‐deleted, granulocyte‐macrophage CSF (GM‐CSF)‐armed attenuated Type‐1 herpes simplex virus that selectively replicates within tumor cells; induces immunogenic cell death; and releases GM‐CSF to promote DC maturation and cross‐presentation of tumor antigens [126]. Combined with pembrolizumab in advanced/metastatic sarcoma, an overall ORR of 35% was achieved within 24 weeks, with a median duration of response of 56.1 weeks and no significant treatment‐related toxicities [127]. In the Phase II TNT trial (T‐VEC plus nivolumab and trabectedin), 11 evaluable LMS patients exhibited a ≤+20% change in the sum of target‐lesion longest diameters at Week 6, with one uLMS case reaching −20%, indicating an early tumor‐shrinkage trend in this subtype [128]. While the individual contribution of each agent remains undetermined, the high DCR observed in LMS justifies initiation of a randomized Phase III study. Future trials should adopt factorial or de‐escalation designs—such as T‐VEC versus no T‐VEC or trabectedin‐free arms—with co‐primary endpoints of PFS and immune microenvironment dynamics, to dissect the independent and synergistic effects of the triplet regimen.

In parallel with T‐VEC, the third‐generation oncolytic herpes simplex virus T‐01 also showed good cytotoxicity and replication ability, sparing normal cells while directly killing tumor cells and enhancing tumor immunity, effectively inhibiting tumor growth in an LMS model, providing a new therapeutic line of thought for refractory sarcomas [129].

Adenovirus‐based oncolytic viruses are also under active development. The Ad5‐derived agent AdAPT‐001 carries a dual‐engineered genome—an E1A‐promoter microdeletion for tumor‐selective replication and replacement of E1B‐19K with a TGF‐β trap transgene—allowing rapid intratumoral amplification and local neutralization of the immunosuppressive cytokine TGF‐β. In the first‐in‐human Phase I trial, four heavily pretreated patients with advanced LMS (median 7, up to 12 prior lines) received intratumoral injections of 1 × 10^12^ viral particles every 2 weeks: one achieved disease stabilization for 6 months, one for 2 months, and two uLMS cases discontinued at approximately 1 month; no grade ≥3 treatment‐related adverse events were observed [130]. These data indicate favorable tolerability of AdAPT‐001 in end‐stage LMS and support the hypothesis that TGF‐β blockade can “heat” immune‐desert tumors, providing a rationale for its ongoing Phase II evaluation in combination with ICIs (NCT04673942).

### 4.5. Other Promising Treatments for LMS

Beyond the immune‐based modalities discussed earlier, several strategies—largely untested but readily transferable to LMS—are now attracting preclinical and early‐phase attention. The tumor‐penetrating peptide iRGD escorts T cells, nanoparticles, or small‐molecule drugs into deep tumor regions. Its affinity for αvβ3/αvβ5 integrins and subsequent CendR‐mediated transcytosis increases intratumoral T cell infiltration by nearly 10‐fold and augments antitumor activity with minimal systemic exposure [131–134]. CRISPR–Cas9 platforms enable multiplex editing in preclinical models: knockout of CD47 or iIFN‐β receptor combined with IL‐12 or GM‐CSF cassettes converts macrophages from an M2 to M1 phenotype, restores DC cross‐presentation, and achieves durable regression of orthotopic xenografts, providing an off‐the‐shelf path toward neoantigen‐agnostic, gene‐edited cell immunotherapy [135, 136]. CRISPR–Cas9 gene editing technology lays a foundation and offers new ideas for LMS therapy.

Finally, immunoradiotherapy is rapidly repositioning conventional radiobiology: Low‐dose fractionation or single high‐dose ablation plus postablative low‐dose “PAM” triggers cGAS‐STING–dependent IFN‐I release, generates an in situ vaccine effect, and synergizes with checkpoint blockade; multisite or node‐sparing stereotactic protocols are under investigation to balance systemic immune activation with normal‐tissue preservation [137–141]. These orthogonal strategies collectively offer new, mechanistically distinct options that are highly promising for advanced LMS.

In summary, ICIs have brought new therapeutic hope to LMS patients, but the overall TME of LMS still presents a “cold tumor” state, and recurrent immune resistance needs to be addressed. In pathology‐reported studies, acquired tumor resistance mechanisms are often associated with neoantigen loss, altered antigen presentation, or disrupted immune signaling [142–145]. While the emerging immunotherapies outlined in this chapter are highly promising, their evidentiary maturity and research volume remain below those of ICI‐focused investigations. The lack of relevant cellular targets and checkpoints, the need for more optimized therapeutic regimens to achieve higher response rates in LMS, and the combination of these issues have spurred the development of many emerging immunotherapies.

## 5. Conclusions

ICI monotherapy consistently fails to produce clinically meaningful responses in LMS, underscoring the need to move beyond single‐agent checkpoint blockade. Emerging evidence indicates that the greatest therapeutic gains are currently achieved with combination regimens, particularly ICI plus trabectedin (SAINT trial: 89.5% DCR, 31.6% ORR, and 36.1‐month median OS in LMS) and ICI plus selected TKIs (e.g., anlotinib or sunitinib), which simultaneously relieve immune suppression and enhance T cell trafficking. Realization of these benefits will require prospective, biomarker‐integrated trials that allocate patients based on validated predictors such as TLS, PD‐L1 expression, *PTEN* loss, or CSF1‐driven macrophage infiltration. Future efforts should prioritize biomarker‐integrated randomized trials, rational combination strategies, and translational research that seamlessly bridges preclinical insights to clinical application, accelerating the delivery of precision immunotherapy for advanced LMS.

**Nomenclature**
ACT: Adoptive cell therapy
CAR‐T: Chimeric antigen receptor T cells
CIK: Cytokine‐induced killer
CR: Complete response
CSF1: Colony‐stimulating factor 1
CTL: Cytotoxic T lymphocytes
CTLA‐4: Cytotoxic T lymphocyte antigen‐4
DC: Dendritic cell
DCR: Disease‐control rate
DFS: Disease‐free survival
GM‐CSF: Granulocyte‐macrophage colony‐stimulating factor
ICIs: Immune checkpoint inhibitors
IFN‐β: Interferon‐β
IL‐2: Interleukin‐2
LAG‐3: Lymphocyte activation gene‐3
LMS: Leiomyosarcoma
M‐CSF: Macrophage colony‐stimulating factor
MAGE: Melanoma‐associated antigen
MHC: Major histocompatibility complex
NK: Natural killer
ORR: Objective response rate
OS: Overall survival
OSM: Oncostatin M
PAM: Postablation modulation
PD‐1/PD‐L1: Programmed cell death protein 1/programmed death ligand 1 blockade
PFS: Progression‐free survival
PR: Partial response
RFS: Recurrence‐free survival
SD: Stable disease
si‐TCR: Small‐interfering RNA
sgRNA: Small‐guide RNA
SIRPα: Signal‐regulating protein α
STS: Soft‐tissue sarcomas
TAAs: Tumor‐associated antigens
TAMs: Tumor‐associated macrophages
TCR‐T: T cell receptor–T cell
TEMRA: CD45RA effector memory T cells
TIL: Tumor‐infiltrating lymphocytes
TLS: Tertiary lymphoid structures
Tregs: Regulatory T cells
TKIs: Tyrosine kinase inhibitors
TMB: Tumor mutation burden
TME: Tumor microenvironment
TSAs: Tumor‐specific antigens
uLMS: Uterine leiomyosarcoma.

## Author Contributions

Haoyue Qi collected relevant literature and received and drafted the manuscript and summarized the figures and tables. Hanxi Zhang, Qin Wang, Xiaolu Wang, Limei Min, Yingling Zhou, Baorui Liu, and Rutian Li reviewed the manuscript and revised it.

## Funding

This study was National Natural Science Foundation of China (Grant 82103687), the Nanjing Medical Science and technology development Foundation (Grant YKK23077), the Foundation for Studies of Clinical Trials, Drum Tower Hospital (Grant 2022‐LCYJ‐MS‐09), and the “333 Project” of Jiangsu Province (Grant (2020)2‐020).

## Disclosure

All authors contributed to the manuscript and approved its submission.

## Consent

The authors have nothing to report.

## Conflicts of Interest

The authors declare no conflicts of interest. Figure 1 was drawn by Figdraw (www.figdraw.com).

## Data Availability

Data sharing is not applicable to this article as no datasets were generated or analyzed during the current study.
